# Editorial: Population Neuroscience of Development and Aging

**DOI:** 10.3389/fnsys.2022.897943

**Published:** 2022-04-25

**Authors:** Tomáš Paus, Stephanie Debette, Sudha Seshadri

**Affiliations:** ^1^Departments of Psychiatry and Neuroscience, Faculty of Medicine and Centre Hospitalier Universitaire Sainte-Justine, University of Montreal, Montreal, QC, Canada; ^2^Departments of Psychology and Psychiatry, University of Toronto, Toronto, ON, Canada; ^3^University of Bordeaux, Inserm, Bordeaux Population Health Research Center, Team VINTAGE, UMR 1219, Bordeaux, France; ^4^CHU de Bordeaux, Department of Neurology, Bordeaux, France; ^5^Department of Epidemiology and Biostatistics, Glenn Biggs Institute for Alzheimer's and Neurodegenerative Diseases, UT Health San Antonio, San Antonio, TX, United States

**Keywords:** brain development, brain aging, cohort studies, genetics, epidemiology, neuroimaging, psychiatric and neurologic disorders

Brain development and aging involve many inter-related biological processes that are shaped by genes and the environment from conception onwards. Population neuroscience endeavors to identify and model such processes and influences using a combination of epidemiology, “omics” sciences and neuroimaging, as applied in large cohorts and meta-analytical datasets. In these efforts, practitioners of population neuroscience are cognizant of three key challenges inherent in their pursuits: (1) An infinite combination of factors influencing the brain from *within* (genes and their regulation) and the *outside* (physical, built and social environment); (2) Presence of *developmental cascades* that carry such influences from one time point to the next, from one organ to another, and from one level of organization to a different one; and (3) Structural and functional *complexity* of the human brain (Paus, 2013, 2016).

This Research Topic collected contributions that speak to the current advancements in relevant methodological and conceptual issues, as well as reviews and original reports drawing on population-based studies of brain development and aging.

On the “development” side of the lifespan continuum, four papers report original findings obtained in four large cohorts of children, adolescents and young adults, namely the Generation R, ABCD Study, IMAGEN and iSHARE. Lopez-Vicente et al. analyzed data acquired during resting state with functional magnetic resonance imaging (MRI) in ~ 3,000 children (8–15 years of age), and provided a glimpse of developmental changes in the dynamics of “functional connectivity” during this developmental period. Patel et al. studied the relationship between various structural properties of the cerebral cortex in ~11,000 10-year old children, and reported a striking similarity between inter-regional profiles of the tangential growth of the cerebral cortex (i.e., its surface area) as it relates—in a distinct spatial pattern captured by the profiles—to both the general psychopathology and overall cognitive abilities. Penninck et al. tested, in over 1,500 adolescents, whether psychopathology at 18 years of age could be predicted by inflammation-related variations in brain structure at 14 years of age; they complemented this work in humans by experimental studies in mice. Finally,  Tsuchida et al. showed, in a cohort of ~1,700 university students (18–26 years of age), that the maturation of structural properties of white matter, characterized by multi-modal imaging, continue to change in the 3rd decade of life, with considerable variations across MRI-based parameters (e.g., fractional anisotropy, neurite density index) and fiber tracts.

On the “aging” side of the lifespan continuum, this Research Topic includes four papers that address a variety of questions about the aging brain. Lin et al. used a modest lifespan sample (417 individuals, 21–92 years of age), and identified striking age-related changes in cortical thickness and curvature in older (60+ years) adults that varied as a function of their location on the surface (gyri) vs. depth (sulci) of cortical folds. Rodriguez et al. studied 1,400 adults (40–80 years of age) to find out whether the type of employment (and associated mental demands) protect brain health, with an affirmative answer especially in individuals who continued to work in their older age. Sole-Padulles et al. examined, in a multi-center study (~1,400 adults over 60 years of age), whether or not loneliness is associated with cognitive and brain health, and concluded that this is not the case if one excludes individuals who developed dementia at follow-up assessments. Finally, Xiang et al. analyzed a unique sample of 250 centenarians to assess the interplay between Apolipoprotein E (*APOE*) genotypes and education vis-à-vis cognitive decline, confirming the beneficial effect of education in carriers of the e3 allele but also revealing some additional, less expected, *APOE*-education interactions.

The above empirical reports are complemented by a series of methodological, conceptual and review papers. Thus, Balsor et al. provided a practical guide for studying the molecular development, across the lifespan, of the human visual cortex, addressing the key challenge of limited *post mortem* proteomic and transcriptomic data. Soumaré et al. addressed the important questions of incidental findings on MRI scans acquired in community-based samples of young adults, and provide a breakdown of the observed findings to those that required medical referral, active intervention, and clinical surveillance. Ness and colleagues asked to what extent developmental trajectories in brain structure and function, as obtained in large population-based samples, relate to symptom manifestations, and how to integrate other sources of data (e.g., social environment) to improve relevant predictions and risk calculations (Nees et al.). Abuga et al. provided a systematic review aimed at estimating the magnitude of premature mortality following childhood-onset neurological impairment (e.g., epilepsy), and identified several risk factors associated with premature death in these children in both high-income and low/middle-income countries. Finally, Royse et al. carried out a qualitative review to determine whether population ancestry (African American, Hispanic, non-Hispanic whites) plays a role in dementia-associated pathology and found that there are significant differences as a function of both ancestry and sex in the case of Tau (but not Aβ) load; given the limited literature, no conclusions could be drawn about the possible interaction between ancestry and sex.

The above contributions are but a few papers addressing important questions about the development, maturation and aging of the human brain using the population-neuroscience framework. This type of work has been enabled by several developments in the field, including (1) the pooling of existing datasets facilitated by collaborative efforts of several international consortia, such as CHARGE and ENIGMA, (2) the establishment of large community-based cohorts, such as the UK Biobank and the ABCD Study, and (3) open-science initiatives in the “omics” sciences, especially those in transcriptomics and proteomics (e.g., Allen Human Brain Atlas, the PsychENCODE Project). We and others have used these resources to answer a variety of questions about the development of the human cerebral cortex across the lifespan (Vidal-Pineiro et al., 2020; Bethlehem et al., 2022), its genetic architecture (Grasby et al., 2020; Hofer et al., 2020; Shin et al., 2020), cellular underpinning of inter-regional profiles of MRI-based phenotypic variations (Shin et al., 2018; Patel et al., 2021), and their prenatal origin (Patel et al., 2022). Now that this Research Topic is completed, we will endeavor to facilitate future inter-disciplinary cross-talks by publishing papers of this and similar Research Topics in the Section on Population Neuroimaging of Frontiers in Neuroimaging, a new Frontiers journal launched this year.

## Author Contributions

TP drafted the manuscript. SD and SS reviewed it. All authors contributed to the article and approved the submitted version.

## Conflict of Interest

The authors declare that the research was conducted in the absence of any commercial or financial relationships that could be construed as a potential conflict of interest.

## Publisher's Note

All claims expressed in this article are solely those of the authors and do not necessarily represent those of their affiliated organizations, or those of the publisher, the editors and the reviewers. Any product that may be evaluated in this article, or claim that may be made by its manufacturer, is not guaranteed or endorsed by the publisher.
